# Editorial: Clinical Guidelines and Real Practice of Antimicrobial Pharmacotherapy

**DOI:** 10.3390/antibiotics15090890

**Published:** 2026-09-11

**Authors:** Darko Modun

**Affiliations:** Department of Pharmacy, University of Split School of Medicine, 21000 Split, Croatia; darko.modun@mefst.hr

Clinical guidelines for antimicrobial pharmacotherapy are crucial tools, built upon extensive research and expert consensus, designed to optimize the treatment of infections. They outline best practices for selecting effective drugs, determining appropriate dosing, and defining treatment duration to standardize care and minimize antimicrobial resistance. However, translating these guidelines into real-world clinical practice presents significant challenges. Variability in patient populations, complex comorbidities, restricted drug availability, and the dynamic nature of microbial resistance patterns frequently necessitate deviations from standardized protocols.

To achieve clinical success, practitioners must adopt a flexible, individualized approach that balances strict adherence to guidelines with the nuanced needs of their patients and local epidemiological data. This Special Issue was established to explore these complexities, and it is my pleasure to summarize the outstanding contributions published herein.

The seven articles published in this Special Issue address various facets of antimicrobial pharmacotherapy, ranging from diagnostic optimization to the management of highly resistant pathogens:

Accelerating Diagnostics: Edmondson et al. demonstrated that reducing post-blood culture incubation for Antimicrobial Susceptibility Testing (AST) from 24 h to 6 h yielded a 99.65% agreement in susceptibility readings, enabling significantly faster targeted therapy for bacteremia without automation [1].
Adapting to Local Resistance Profiles: Enciu et al. highlighted a high prevalence of multidrug-resistant (MDR) and extended-spectrum beta-lactamase (ESBL)-producing strains in acute complicated diverticulitis, emphasizing that empiric therapies must be strictly tailored to local resistance profiles to prevent treatment failure [2].
Optimizing *H. pylori* Eradication: Gelley et al. showed that supplementing 14-day clarithromycin-based regimens with bismuth and probiotics achieved high cure rates in a clarithromycin-resistant region, serving as a highly effective alternative to first-line tetracycline-based therapies when availability is limited [3].
Vaccination and Resistance Trends: Bondi et al. explored the epidemiology of *Streptococcus pneumoniae*, observing an increased risk of penicillin G-resistant invasive pneumococcal disease among infants and the elderly following the introduction of specific anti-pneumococcal vaccines, while noting that the COVID-19 pandemic had no significant impact on resistance rates [4].
MRSA Biofilms in Endocarditis: Kaushik et al. comprehensively reviewed the complexities of treating infective endocarditis caused by biofilm-producing methicillin-resistant *Staphylococcus aureus* (MRSA), highlighting the urgent need for novel therapies—such as bacteriophages or antimicrobial peptides—to disrupt these highly protective bacterial matrices [5].
Beta-Lactamase Inhibition Dynamics: Wang et al. utilized dynamic hollow-fiber infection models to confirm that sulbactam, when combined with ceftriaxone, exerts a sustained post-beta-lactamase-inhibiting effect against ESBL-producing *Escherichia coli*, successfully maintaining enzyme inhibition even after sulbactam elimination [6].
Repurposing Antimicrobials: Radić et al. examined the dual anti-inflammatory and immunomodulatory properties of tetracyclines in the context of rheumatoid arthritis, noting that while mechanistically promising, their clinical integration remains limited by concerns over antimicrobial resistance [7].

As we officially close the “Clinical Guidelines and Real Practice of Antimicrobial Pharmacotherapy” Special Issue, it is evident that the future of infectious disease management lies in personalized medicine, rapid diagnostics, and rigorous regional stewardship. I extend my deepest gratitude to all the authors, peer reviewers, and the editorial team at *Antibiotics* for their dedication and invaluable contributions to this successful collection.
